# Contrasting impacts of *Plantago ovata* fibre fractions on corn starch structure and digestibility

**DOI:** 10.1039/d5fo02366a

**Published:** 2025-10-27

**Authors:** Lucija Štrkalj, Rachel A. Burton, Gleb E. Yakubov, James M. Cowley

**Affiliations:** a School of Agriculture, Food and Wine, University of Adelaide Waite Campus Urrbrae SA Australia; b Division of Food, Nutrition and Dietetics, School of Biosciences, University of Nottingham Sutton Bonington Campus Loughborough Leicestershire UK lucija.strkalj1@nottingham.ac.uk; c Food Biopolymers Laboratory, School of Food Science and Nutrition, University of Leeds Leeds LS2 9JT UK

## Abstract

Psyllium husk, a dietary fibre derived from *Plantago ovata* seeds, is widely used in food systems for its gelling ability, water absorption, and texturizing properties. Due to its varying solubility, the fibre can be extracted into fractions with different flow properties. However, limited knowledge exists on how its fractions can be leveraged for food enhancement. Therefore, we isolated two psyllium fractions (F1 and F2) and fabricated starch-fibre gels using starches with varying amylose content (5.9, 37.9, and 63.1% amylose). To fully understand mechanisms between the fractions and starches, we applied three temperature treatments (95, 120 and 140 °C) by utilising high temperature rapid visco analysis (HT-RVA), resulting in 27 starch and starch-fibre gels. F2 increased peak viscosity in all starches, and it significantly changed the profile of high amylose corn starch, while F1 had less effect on the pasting. Texture properties of the gels were mostly influenced by temperature treatments, but amylose leaching and starch hydrolysis by α-amylase were significantly changed by fibre addition. F1 caused an increase of the extent of starch hydrolysis, but F2 reduced it. F1 increased leached amylose, particularly in high amylose corn starch. SEM images have shown changes in the gel microstructure depending on fibre addition and temperature, potentially related to phase separation and fibre impact on ice formation. This work highlights that fibre fractions from the same source have contrasting effects on functional and health-related properties of starch-based food systems, which may be highly valuable for developing healthier food products.

## Abstract 2

This study demonstrates that distinct psyllium fractions differentially modulate starch gel functionality, microstructure, and health-related properties, enabling targeted food innovation.

## Graphical abstract

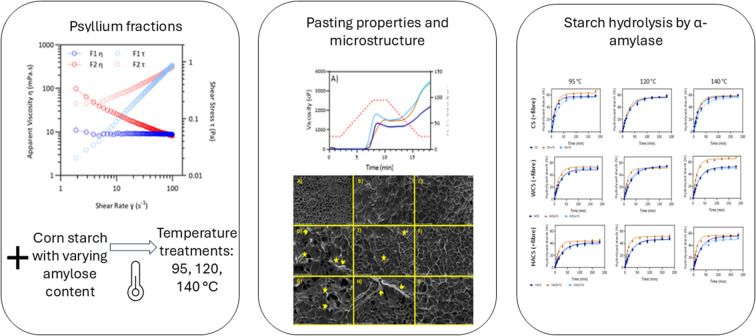

## 1. Introduction

Starch is estimated to have the largest contribution to energy intake worldwide.^1–3^ In addition to staple foods like bread, rice, and pasta, starch is implemented into an array of food products as there are number of source options, associated variation in functionalities, and the ease of application. In practice, starch hydrogels are commonly used for assessing the techno-functional properties of starch-containing products. They are inexpensive and can be used in conjunction with many experimental techniques, whilst retaining close relationship with in-food behaviours of starches.^4^ Starch gels are produced by mixing starch with water and heating the mixture; upon cooling a gel-like consistency is achieved. Starch properties after cooking determine the quality of final product such as structure, digestibility, and storage stability. While starch has a pivotal role in food production and nutrition research, it is essential to recognize its shortcomings, as well. Storage stability can present a marked challenge, as starch gels can be prone to syneresis under lower temperatures, which influences sensory perception and product structure.^5,6^ Moreover, starchy foods can cause spikes in blood glucose levels (glycaemia), leading to higher risks of developing metabolic diseases.^7^

The addition of non-starch polysaccharides (NSP), such as fibre and gums can improve structure and texture, storage stability, and digestibility of starch gels. Generally, NSPs delay starch gelatinization and reduce digestibility through different mechanisms; NSPs can form a barrier around starch granules and restrict amylases’ access to the substrate; in addition, they can compete for water and thus reduce starch swelling and gelatinization. Finally, NSPs can non-competitively bind to α-amylase and act as a decoy substrate, thereby reducing the rate of hydrolysis.^8^ For example, addition of pectin from citrus peel (rhamnogalacturonan-I) to potato starch decreased hardness of the gel and increased its slowly digestible starch content.^9^ Heteroxylan from corn fibre decreased retrogradation and led to more stable corn and wheat starch gels.^10^ Wheat arabinoxylan delayed long-term retrogradation and increased hardness when added to wheat starch gel.^11^ It has also been shown that certain types of pectin with low degree of esterification decreased maize starch digestibility by non-competitive inhibition of α-amylase.^12^ Moreover, addition of fibre in starch-based products is perceived as “clean and healthy” by the wider population, as opposed to using modified starches. Overall, research has shown that NSP addition can have a profound effect on the quality of starch gels, such as delay of retrogradation, storage stability and texture and structure improvement.^4^

A source of NSP fibre of particular interest to our research groups is *Plantago ovata* Forssk., widely known as psyllium.^13–15^ Psyllium husk is produced from the milled coating of *P. ovata* seeds. When in contact with water, the husk swells and forms a strong gel with complex structure.^13,15^ It is widely used in food industry, as an additive to conventional bakery products, gluten-free bread, yoghurts, jams, and can be used as fat replacer, stabiliser, and texturiser.^16^ It has numerous health benefits, such as cholesterol reduction, satiety and appetite regulation, gastrointestinal relief, and reduction in postprandial glucose.^17^ When incorporated into wheat, potato, or tapioca starch, psyllium can reduce rapidly digested starch and increase slowly digested starch.^18^ When comparing psyllium and xanthan gum properties in maize starch gels, it was reported that both NSPs reduced hardness, while psyllium changed pasting properties.^19^

Due to a rising interest in *P. ovata* fibre gel structure, different extraction protocols have been developed in order to accommodate its unique rheological (flow) properties and high structural complexity.^15,20–22^ It was confirmed that fractions extracted with H_2_O at lower temperature (up to 25 °C) forms viscoelastic fluid and consists of heteroxylan with a minor (>10%) pectin component. Fractions subsequently extracted at higher temperatures (65 °C and higher), with alkaline solvents (2 M KOH), or *via* intense shaking, were found to consist of highly branched heteroxylan (depleted of pectin) which forms significantly stronger gels.^15,20–24^ Detailed rheology and structure studies of different fractions have helped elucidate complex structures and functional behaviours of psyllium gels, however there is a lack of practical application of said fractions. Moreover, there is a gap in understanding how *P. ovata* fibre and particularly fractionated fibre affects starch gels and starch-based products. Recently, Govender and Amonsou reported on the incorporation of 3% psyllium into various corn starches.^25^ Although their study also employed high-temperature RVA analysis, their sample preparation procedures differed substantially from ours, and they utilized whole psyllium husk.

Therefore, since psyllium fractions exhibit apparent flow behaviour, this study focuses on investigating whether starches combined with different fractions, rather than the whole husk, display distinct techno-functional properties. To the best of our knowledge, no work was published about incorporation of psyllium fractions to starch gels and their effect on pasting profile of corn starch. Therefore, this complex gel-forming NSP presents an opportunity to probe the impact of distinct fibres in starch systems.

In this work, a simple extraction protocol was used to obtain two different fibre fractions from the same source (commercial psyllium husk). The practical application of said fibres was assessed on three corn starches under three temperature conditions. The structure, techno-functional properties, and digestibility of fibre-starch gels were explored. In this study, we prioritized examining the outcomes over elucidating the underlying mechanisms for the aim of studying how distinct fibre additions and processing conditions affect different starches, and how this knowledge can be translated into digestibility and formulation of new food products. Nevertheless, the reported results provide a basis for continuing further study of underpinning mechanisms of interactions between starch and *Plantago* fibre fractions.

## 2. Materials and methods

### 2.1. Materials

Three types of starch were used: corn starch (Sigma Aldrich, S4126), waxy corn starch (VPA, Queensland), and high amylose corn starch (Hylon™ VII, Ingredion††HYLON and INGREDION are trademarks of Ingredion Incorporated., New South Wales). For starches, we use following abbreviations: CS = corn starch, WCS = waxy corn starch, HACS = high amylose corn starch.

Psyllium husk (Bonvit, Woolworths, South Australia) was used for extraction of two fibre fractions. An 8 g aliquot of husk was dispersed in 800 mL RO H_2_O and stirred (400 rpm) for 4 h at 4 °C. The mixture was centrifuged at 3600 rpm (30 min, 4 °C) (5810R centrifuge, Eppendorf, New South Wales). The upper layer (fraction 1) was separated from the lower part (fraction 2). The upper fraction (supernatant) was a slightly viscous liquid, while lower fraction (pellet) was a gel with solid husk remnants (visually determined). Both fractions were separately dried overnight in an air oven at 95 °C. After drying, fibres were ground in coffee grinder or milled in a mixer mill (Retsch MM400, Germany) (75% filling, 10 min, 30 Hz) and sieved using 200 μm sieve. For fibre fractions we use following abbreviations: F1 = dried and sieved supernatant, F2 = dried and sieved pellet.

Porcine pancreatic α-amylase (E-PANAA, 75 000 U g^−1^) was purchased from Megazyme (Neogen Australasia, Queensland, Australia). Amylose from potato (A0512), d-maltose monohydrate (63 418), 4-hydroxybenzhydrazide (H9882), trichloroacetic acid (T0699), sodium carbonate (223 530), iodine (207 772), and sodium phosphate monobasic (S0751) were purchased from Sigma Aldrich (New South Wales, Australia). Sodium phosphate dibasic (SA026), potassium iodide (PA001), and sodium hydroxide (SA000) were purchased from ChemSupply (South Australia). Hydrochloric acid (RP1104) was purchased from RCI Labscan (Victoria). Amylopectin (A0456) from waxy corn was purchased from Chem-Supply (Gillman, South Australia). All used chemicals were of analytical grade.

### 2.2. Methods

#### 2.2.1. Characterization of starches and fibres

Apparent amylose content of starches was determined by 96-well plate assay developed by Kaufman *et al.* without any modifications.^26^ Particle size distribution of starches was carried out using a multi-laser Particle Size Analyser (PSA1190, Anton Paar, Austria) using a dispersion air setting of 4000 mbar. The internal Kalliope software calculated the particle size distribution on a volume basis. Results are means of duplicate runs.

Fibre yield was determined by weighing the mass of psyllium before extraction process, and weighing the masses of F1 and F2 after drying.1
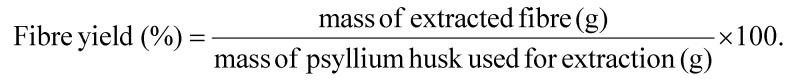


FTIR spectra of powdered F1 and F2 were collected using a Nicolet iS20 FTIR spectrometer (Thermo Scientific, USA) using 48 scans at a resolution of 4 cm^−1^. Monosaccharide composition of F1 and F2 was determined by using reverse phase high performance liquid chromatography (RP-HPLC) of 1-phenyl-3-methyl-5-pyranozile (PMP) derivatives following the process described in Hassan *et al.* with modifications specified in Cowley *et al.* where the fibre was pre-dispersed at 1 mg mL^−1^ in hot water.^20,27^ Area under the peaks was compared to standard curves of mannose, ribose, rhamnose, glucuronic acid, galacturonic acid, glucose, galactose, xylose, arabinose and fucose.

Water retention capacity (WRC) and water swelling capacity (WSC) of F1 and F2 were assessed according to method by Noguerol *et al.* with no modifications.^28^ Viscosity of 0.4% dispersions of F1 and F2 were determined using an air-bearing rheometer (HAAKE MARS iQ Air, Thermo Fisher Scientific, Waltham, United States). First, dispersions were prepared using a Rapid Visco Analyser (RVA 4800, Perten Instruments, New South Wales). Powdered F1 or F2 (120 mg) were added to 30 mL of distilled water, briefly mixed by vigorous stirring, then placed into the RVA. The samples were heated from 25 °C to 95 °C over 5 minutes, held at 95 °C for 5 minutes, then cooled to 25 °C over 5 minutes. Paddle speed was 960 rpm during the first minute, and 160 rpm during the rest of the protocol. After preparation, the samples were homogenised for 60 seconds at 6000 rpm using a PROScientific Premium MaX homogenizer (PRO Scientific Inc., CT, USA) and then sealed to prevent evaporation. Samples were stored at room temperature for 3 hours before viscosity testing. Viscous behaviour was assessed using a shear rate ramp from 0.1–100 s^−1^ over 5 min (after a 2 min equilibration) with a concentric cylinder system (HAAKE CCB25 DIN/SS and CC25 DIN/Ti, Thermo Fisher Scientific, Waltham, United States, gap between the cylinders 0.622 mm) held at 20 °C with a Peltier unit (TM-PE-C, Thermo Electron GmbH, Karlsruhe, Germany) controlled by a heat exchanger (HAAKE HX-R, Thermo Fisher Scientific, Waltham United States). The power-law fluid (Ostwald–de Waele) model was used to evaluate the rheological properties of the dispersions (eqn (2)). Modelling was performed in GraphPad Prism 10.4.0.2
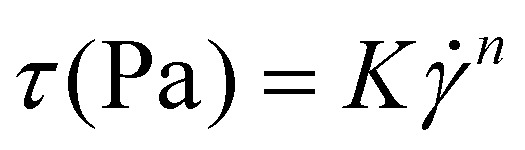
where *τ* refers to the shear stress in Pa, 
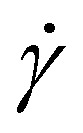
 represents the shear rate in s^−1^, *K* is the flow consistency coefficient and *n* is the dimensionless flow behaviour index. From the models, the viscosity at a shear rate of 10 s^−1^ (Visc^γ10^) was also interpolated for comparison.

#### 2.2.2. Pasting behaviour

Rapid Visco Analyser (RVA 4800, Perten Instruments, New South Wales) was used to simultaneously prepare and assess the pasting behaviour of starch and starch-fibre gels. A 1.875 g (dw) aliquot of starch was weighed into the aluminium RVA canister and H_2_O was added to a total mass of 25 g. When preparing starch-fibre gels, 0.1 g of fibre was added. The final mass was 25.1 g. Addition of fibre based on the total gel mass and the starch mass was 0.4% and 5.3%, respectively. For the preparation of fibre–starch gels, fibre and starch (both in their dry forms) were premixed prior to the addition of water, after which the RVA protocol was initiated. This procedure was applied to all starch samples. By combining fibre and starch in the dry state before hydration, both components were provided with an equal opportunity to hydrate during subsequent processing. Three RVA protocols were used based on maximum temperature (*T*_max_) reached during pasting: 95, 120, and 140 °C. To ensure all samples were treated at corresponding *T*_max_ for equal time period and rate of heating/cooling was equal (12.3 °C min^−1^), protocols had varying time lengths. Therefore, all protocols followed the same outline (hold at 25 °C for 2 min → increase temperature to *T*_max_ → hold at *T*_max_ for 2.5 min → decrease temperature back to 25 °C → 25 °C hold at for 2 min), but the lengths of the runs were: *T*_max_ 95 °C–18.10 min, *T*_max_ 120 °C–22.20 min, and *T*_max_ 140 °C–25.40 min (detailed scheme for RVA protocols are shown in SI Fig. S1). Paddle speed was 960 rpm during the first minute, and 160 rpm during the rest of the protocol. Gels from the RVA were then used for further characterisation. In Table 1, we specify the sample preparation and abbreviations which will be used throughout the rest of this work.

**Table 1:** List of starches and fibres treated at specified temperature to prepare the gels and the corresponding sample name

Starch	Fibre	Temperature treatment (°C)	Sample name
Corn starch	—	95	CS-95
Corn starch	F1	95	CS+F1-95
Corn starch	F2	95	CS+F2-95
Corn starch	—	120	CS-120
Corn starch	F1	120	CS+F1-120
Corn starch	F2	120	CS+F2-120
Corn starch	—	140	CS-140
Corn starch	F1	140	CS+F1-140
Corn starch	F2	140	CS+F2-140
Waxy corn starch	—	95	WCS-95
Waxy corn starch	F1	95	WCS+F1-95
Waxy corn starch	F2	95	WCS+F2-95
Waxy corn starch	—	120	WCS-120
Waxy corn starch	F1	120	WCS+F1-120
Waxy corn starch	F2	120	WCS+F2-120
Waxy corn starch	—	140	WCS-140
Waxy corn starch	F1	140	WCS+F1-140
Waxy corn starch	F2	140	WCS+F2-140
High amylose corn starch	—	95	HACS-95
High amylose corn starch	F1	95	HACS+F1-95
High amylose corn starch	F2	95	HACS+F2-95
High amylose corn starch	—	120	HACS-120
High amylose corn starch	F1	120	HACS+F1-120
High amylose corn starch	F2	120	HACS+F2-120
High amylose corn starch	—	140	HACS-140
High amylose corn starch	F1	140	HACS+F1-140
High amylose corn starch	F2	140	HACS+F2-140

#### 2.2.3. Texture profile analysis

An aliquot of 4.5 mL of RVA gels were taken and placed into 5 mL plastic cylindrical tubes. TA.XTplusC Stable MicroSystems texture analyser (Key Diagnostics Pty Ltd, New South Wales) with a small rounded cylindrical probe (4 mm radius, stainless steel with radius end, P/MT, Stable Micro Systems, UK). Triggering force was 1 g, pre- mid- and post-test speeds were 20 mm s^−1^, penetrating to the sample depth of 15 mm (50% of total height) after which the probe returned to original position. Exponent Connect version 8.0.17.0 software was used for data collection, and evaluation of hardness and adhesiveness.

#### 2.2.4. Freeze–thaw syneresis

Freeze–thaw syneresis of RVA gels was done according to Ma *et al.* with some modifications.^29^ Samples were subjected to freeze-thawing cycles for 15 days (22 h at −20 °C, followed by 2 h at 40 °C), after which the upper water layer was carefully removed without centrifugation. Syneresis was calculated as percentage of weight of gel after syneresis divided by weight of gel on day 0 (eqn (3)).3
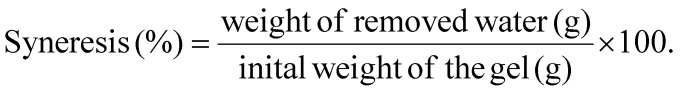


#### 2.2.5. Scanning electron microscopy (SEM)

A thin layer of gel samples from RVA were immediately smeared onto SEM stubs with carbon stickers and then rapidly frozen at −80 °C. After, they were freeze dried at −40 °C for 48 h. Freeze-dried samples were sputter-coated with gold. Scanning electron microscope FlexSEM 1000 (Hitachi, Japan) was used in high-vacuum mode with spot size of 10.0 kV voltage, and FlexSEM 1000 software was used to obtain images. At least three locations were randomly chosen to take micrographs of each sample, and magnification of CS and HACS was 250×, and for WCS it was 150×.

#### 2.2.6. Determination of leached amylose

Amylose leaching assay was adapted from Ma *et al.*^29^ The RVA gels (5 mL) were mixed with 5 mL water and shaken for 2 min (without any ball bearings, 30 Hz) using a mixer mill (MM400, Retsch, Germany). After shaking, the gel was centrifuged for 30 min at 16 000*g* and 25 °C (Eppendorf 5417R, Germany). The supernatant (3 mL) was taken and mixed with 6 mL of 0.33 M NaOH, followed by heating at 95 °C for 30 min. After the mixture was left to cool to room temperature, 100 μL was taken and mixed with 5 mL of 0.5% TCA and 50 μL of 0.05 N I_2_-KI dye. After brief vortexing, mixture was left in the dark to allow for colour development. After 30 min, 300 μL of the solutions was pipetted into a well in 96-well plate with flat bottom. A standard curve was prepared following the same process with a known concentration of amylose. Absorbance was read using plate reader (μQuant, Bio-Tek Instruments, United States) at 620 nm.

#### 2.2.7. Starch hydrolysis by α-amylase and maltose quantification by PAHBAH assay

Starch digestibility by α-amylase was determined based on the work by Edwards *et al.*^30^ The starch to α-amylase ratio is kept constant in all samples, meaning that 100 mg of starch (in starch gel or in starch-fibre gel) was placed into 15 mL tubes and suspended in 10 mL phosphate buffered saline (pH = 7.4). After brief homogenising (PROScientific Premium MaX homogenizer, PRO Scientific Inc., CT, USA) sample tubes were placed in rotary tube mixer inside an incubator (Bioline, Edwards Instrument Company Australia, New South Wales) at 37 °C and equilibrated for 30 min. Before starting the reaction, 100 μL of each sample was transferred into 1.5 mL microcentrifuge tube which contained 100 μL of 0.3 M Na_2_CO_3_ (“stop solution”). The purpose of stop solution is to inactivate the enzyme and stop the reaction at each time point. To start the enzymatic reaction, porcine pancreatic α-amylase was added. Prior to that, the enzyme (75 000 U g^−1^) was carefully weighed and dissolved in PBS to ensure that final enzyme activity was 4.5 AU mL^−1^. Preliminary experiments were carried out to determine the suitable activity. One activity unit is defined as the amount of α-amylase needed to liberate 1.0 mg of maltose from starch in 3 min at pH = 6.9 at 20 °C. Immediately after the addition of the enzyme, the tubes were returned into the incubator and rotated at 37 °C for the rest of the experiment. At pre-defined time points (2, 5, 7, 10, 15, 30, 90, 120, 150 and 180 min) 100 μL aliquots were taken from the reaction solution and placed into microcentrifuge tubes with equal amount of stop solution. After brief vortexing, all tubes were centrifuged at 15 000*g* for 5 min (Eppendorf 5417R, Germany) to exclude any starch remnants, a 100 μL aliquot of supernatant was placed into new microcentrifuge tubes, which were then used to determine the amount of reducing sugars. Reducing sugar analysis (PAHBAH assay) was used to determine the amount of reducing sugars which are the product of α-amylase reaction at each sampled time point.^31^ Supernatants were diluted (1 : 20) in water, and 100 μL of the diluted samples was placed in a heat-safe microcentrifuge tube which contained 1 mL of PAHBAH reagent (preparation: 250 mg of *p*-hydroxybenzoic acid hydrazide dissolved in 4.75 mL of 0.5 M HCl and 45 mL of 0.5 M NaOH). The mixture is vortexed, and then placed in boiling water bath (100 °C) for 5 min. After cooling to room temperature (15 min), 300 μL of each solution was transferred into a well of a flat-bottomed 96-well plate and the absorbance was measured at 405 nm (μQuant, Bio-Tek Instruments, Queensland). Standards containing a known concentration of maltose (concentrations ranging from 100 μM to 1000 μM) were prepared the same way as described above and used for creating the calibration curve against which the samples were measured. The fibre solutions (1% w/v in PBS) were also tested using the PAHBAH assay to ensure there is no absorbance signal, which may interfere with the maltose measurement.

#### 2.2.8. Statistical analysis

Data obtained from the described experiments are presented as the mean and standard deviation of at least triplicates. Starch hydrolysis curves were plotted in OriginPro 2022b software and fitted to first-order equation using non-linear regression, with parameter “*a*” as the extent of hydrolysis (*C*_∞_) and parameter “*b*” as the rate of hydrolysis (constant *k*). Area under curve (AUC) was calculated according to Goñi *et al.*^32^ Two-way analysis of variance (ANOVA) with *post-hoc* Tukey test (0.05 level of significance) was performed in OriginPro 2022b software. Fibres’ properties were statistically analysed by Student's *t*-test with 0.05 level of significance in GraphPad Prism 9.0.0. Statistically significant differences between the samples were marked with different letters, and the samples within the same starch group were compared to each other as the goal of this paper was to compare whether psyllium addition had an impact on the starch gel (*i.e.* corn starch gels with and without psyllium were analysed *via* ANOVA, but they were not compared to waxy corn starch gels or high amylose starch gels). Graphs were made in GraphPad Prism 9.0.0. software, unless otherwise stated.

## 3. Results and discussion

### 3.1. Characterization of starches and fibres

**Fig. 1:**
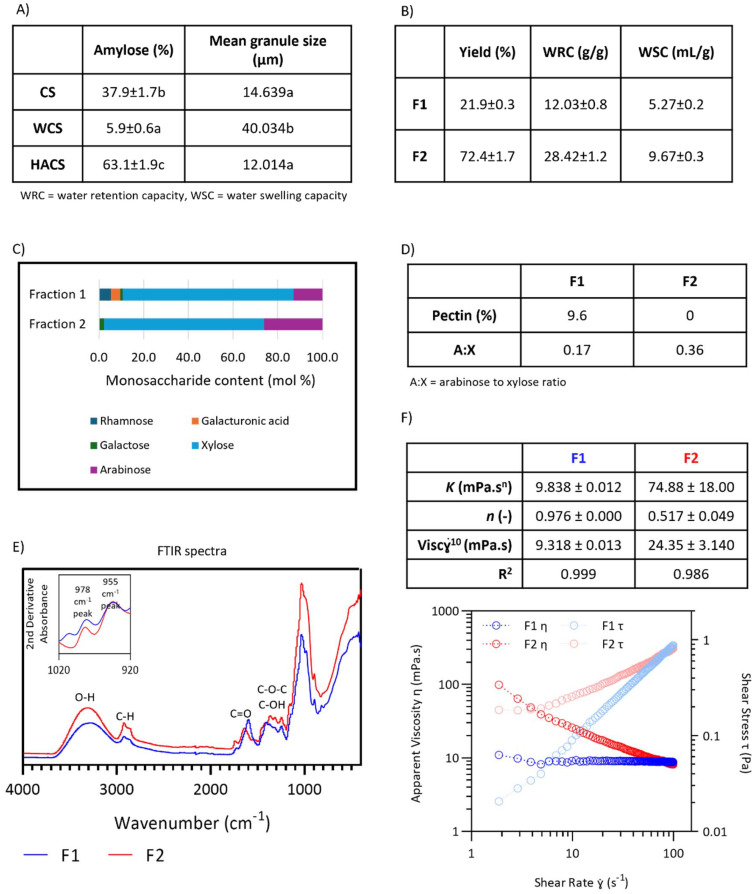
Properties of starches (A – amylose content and mean granule size) and fibres (B – yield, water retention capacity, and water swelling capacity; C – monosaccharide composition, D – pectin content and arabinose to xylose ratio), E – FTIR spectra with 2^nd^ derivative inset, F – apparent viscosity and corresponding coefficients, *K* – consistency coefficient, *n* – flow behaviour index, and interpolation of the viscosity at a shear rate of 10 s^−1^ (Visc^γ10^). The properties (yield, WSC, WRC, and viscosity parameters) of F1 and F2 were tested by Student's *t*-test and are determined to be significantly different at *p* < 0.05.

Characterisation of starch particle size and amylose content are presented in Fig. 1A. CS and HACS had a similar granule size (14.639 and 12.014 μm, respectively), while WCS showed larger granules (40.034 μm). As expected, HACS had the highest amylose content (63.1%), while WCS had the lowest (5.9%). Amylose content of CS was 29.2%, which agrees with the published literature.^33^

The following properties of fibre were determined: yield, water retention capacity, water swelling capacity (Fig. 1B), monosaccharide composition (Fig. 1C), arabinose to xylose ratio (A : X), and pectin content (Fig. 1D), FTIR spectra (Fig. 1E), and steady shear viscosity (Fig. 1F). Both fibres were predominantly heteroxylans, but F1 contained ∼10% pectin (calculated as rhamnose + galacturonic acid). The F2 had a higher A : X ratio than F1 (0.36 and 0.17, respectively), indicating a higher degree of branching. While the linkage composition of the fibre used in this work was not determined, it can be postulated that F2 had higher degree of branching than F1. Yu *et al.* have extracted 3 fractions of *P. ovata* fibre (25, 65 °C, and alkaline extraction) and reported similar monosaccharide composition to ours.^15^ The fractions which have been extracted *via* harsher methods (*i.e.* higher temperature, or alkaline solvents) have shown higher degree of branching and higher A : X ratio. As the branching degree and structural complexity increase, the fibre becomes more difficult to extract, thus requiring more severe conditions for solubilisation and extraction. Ren *et al.* have utilised detailed rheology studies on five fractions from psyllium husk powder and reached the same conclusion as well – the fraction which was extracted at 20 °C showed lower branching than the fractions extracted at 40, 60, 80, and 100 °C, as suggested by the results from FTIR, NMR, and relaxation measurements.^21^ Decreased extractability and increasing gelling capacity were associated with further extraction steps.^15^ The F1 had a much lower yield than F2 (21.9% and 72.4%, respectively). Cowley *et al.* have studied three-step extraction of *P. ovata* mucilage – extraction at 25 °C followed by 65 °C, and lastly, extraction *via* intense shaking.^34^ They reported that the fraction at extracted 25 °C accounted for 31% of extracted mucilage. The F1 had slightly lower yield as it was extracted at 4 °C (21.9% as opposed to 31%), however they do seem comparable. The F2 had a higher water retention capacity (WRC) than F1 (28.42 and 12.03 g H_2_O per g fibre, respectively). This shows that F2 could retain a higher amount of water, which leads to increased swelling. Consequently, water swelling capacity (WSC) were 5.27 and 9.67 mL H_2_O per g for F1 and F2, respectively. Noguerol *et al.* have reported WRC (25.8 g H_2_O per g) and WSC (11.7 mL H_2_O per g) of whole psyllium husk.^28^ Our WRC and WSC values were somewhat higher, which is most likely due to higher degree of fibre purification. It has been reported that psyllium husk powder had water holding capacity of 45.715 g H_2_O per g psyllium powder.^35^ The FTIR spectra of F1 and F2 are very similar and is typical for heteroxylan fibre (Fig. 1E). Broad peak from 3600 to 3000 cm^−1^ represents O–H stretching, followed by smaller peak at ∼2900 cm^−1^ of C–H stretching.^15,21^ A slight difference can be seen in the peak around 1600–1700 cm^−1^ where F1 showed more pronounced peak. This can be attributed to C=O stretching of the carboxyl group in galacturonic acid.^21^ This agrees with the monosaccharide composition which showed that F1 contains galacturonic acid. The cluster of peaks from 1550 to 1100 cm^−1^ can be explained by C–O–C glycosidic bonds vibrations and C-OH stretching.^21^ The FTIR spectra can also shed light on the chemistry of heteroxylan, as evidenced by Ren *et al.*^21^ These authors found that the ratio between peaks at ∼978 cm and ∼955 cm in the second-derivative spectra, were correlated with A : X ratios determined by HPLC and NMR (higher ratios correlated with higher A : X). While these peaks were presented at slightly different wavenumbers, we found the opposite (Fig. 1D, inset), though we only had two fractions and the F2 contained residue. A study on wheat arabinoxylans FTIR spectra showed that highly substituted arabinoxylans had greater peaks at ∼955 cm^−1^ and lower peaks at 985 cm^−1^.^36^ This is consistent with our results as F2 is expected to have higher degree of substituted than F1. Overall, the FTIR results support the monosaccharide analysis (the absence of pectin in F2 and more complex substitution in F2 *vs.* F1). Steady shear viscosity of fibre dispersions confirmed rheological differences between the fractions. F2 was highly viscous when compared to F1 (Fig. 1F). *K*, the flow consistency coefficient, of F1 was 9.838 mPa s, while *K* of F2 was 74.88 mPa s. Moreover, interpolated viscosity at a shear rate of 10 s^−1^ (Visc^γ10^) for F1 was 9.117 and for F2 it was 24.35 mPa s. At the concentrations tested, F1 behaviour is close to Newtonian-like, as it showed consistent viscosity throughout the measured shear range (0–100 s^−1^), with a flow behaviour index *n* near to 1 (*n* = 0.976), while F2 displayed shear-thinning behaviour (*n* = 0.517), most likely due to higher degree of inter-molecular association.

### 3.2. RVA profiles

RVA characterizes starch cooking behaviour by measuring viscosity changes during a controlled heating-holding-cooling cycle.^37^ Pasting profiles reflect key transitions, including granule swelling, gelatinization, rupture, amylose leaching, loss of crystallinity, and eventual amylose reassociation upon cooling.

Here, we should highlight the need for clarification on the use of terminology. The terms “gel” and “paste” are often used interchangeably when discussing starch–H_2_O mixtures. However, there are certain differences. The term “paste” means the hot paste from the RVA, while the term “gel” refers to the cooled (and sometimes stored) paste.^4,38^ A paste is a viscoelastic or viscoplastic fluid which does not stand on its own, while a gel can self-support its structure. In this work, RVA profiles ended at 25° C, thus producing samples at room temperature. All samples could support itself (not shown), except WCS, WCS-F1, and HACS at 95 °C (with and without fibre). To clarify, WCS and WCS-F1 samples are technically pastes, and HACS samples are free-flowing suspensions of starch (and fibre) in H_2_O.

(1) Influence of temperature on pasting characteristics of the three corn starch gels with varying amylose content

**Fig. 2:**
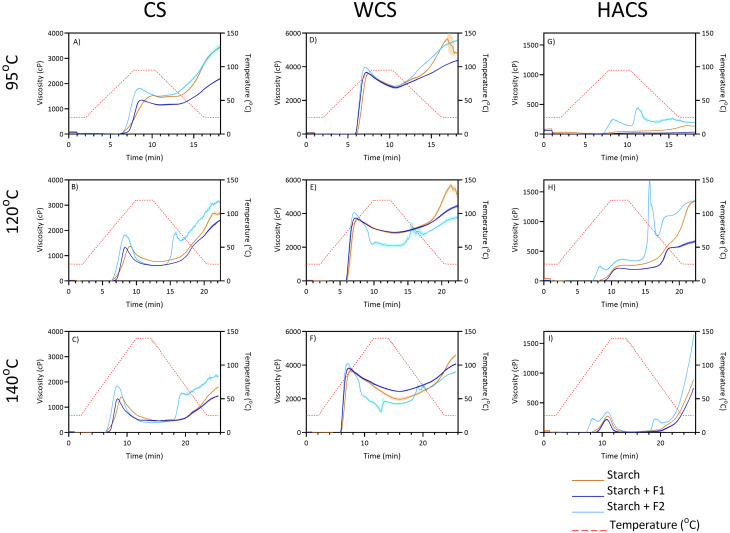
RVA profiles of starches and starch-fibre gels grouped per temperature treatment. Panels A–C show CS gels, panels D–F show WCS gels, and panels G–I show HACS gels. Dark blue line represents starch, brown line represents starch + F1, and light blue line represents starch + F2.

RVA profiles of all gels are presented in Fig. 2, and the parameters are presented in Table 2. Previous papers have been published on HT-RVA treatment on corn starches with varying amylose content.^39,40^ However, we include the starch pasting curves as benchmarks for comparison to understand the effect of F1 and F2 on starches.

**Table 2:** RVA parameters of starch gels and starch-fibre gels. Different letters correspond to significantly different results at *p* < 0.05

		Peak viscosity (cP)	Trough viscosity (cP)	Breakdown viscosity (cP)	Final viscosity (cP)	Setback viscosity (cP)	Time of peak viscosity (min)	Pasting temperature (°C)	Temperature at peak viscosity (°C)
95 °C	CS	1346 ± 3cd	1158 ± 18c	188 ± 18g	2190 ± 17e	1033 ± 25e	8.67 ± 0.05c	84.72 ± 0.18a	94.98 ± 0.03d
	CS + F1	1529 ± 2b	1438 ± 11b	91 ± 12h	3425 ± 38a	1987 ± 27b	10.17 ± 0.18a	76.97 ± 0.92b	95 ± 0d
	CS + F2	1809 ± 17a	1500 ± 13a	309 ± 14f	3460 ± 115a	1960 ± 105b	8.48 ± 0.04de	75.8 ± 0.20b	94.92 ± 0.03d
120 °C	CS	1328 ± 6d	606 ± 4e	721 ± 7d	2393 ± 21d	1786 ± 21c	8.33 ± 0.03de	83.6 ± 0.05a	100.88 ± 0.25bc
	CS + F1	1377 ± 23d	764 ± 5d	608 ± 25e	2678 ± 57c	1915 ± 60bc	9.03 ± 0.02b	75.98 ± 1.02b	109.35 ± 0.26a
	CS + F2	1819 ± 27a	604 ± 13e	1215 ± 14b	3121 ± 62b	2517 ± 75a	8.28 ± 0.02e	75.35 ± 0.56b	100 ± 0.23c
140 °C	CS	13 333 ± 10d	457 ± 10g	876 ± 11c	1446 ± 13g	989 ± 3e	8.37 ± 0.02d	83.92 ± 0.03a	101.3 ± 0.20b
	CS + F1	1404 ± 7c	516 ± 12f	888 ± 5c	1802 ± 21f	1286 ± 9d	9.06 ± 0.04b	75.83 ± 0.85b	109.77 ± 0.44a
	CS + F2	1841 ± 21a	387 ± 14h	1454 ± 13a	2224 ± 77e	1837 ± 78bc	8.30 ± 0.03de	75.52 ± 0.18b	100.52 ± 0.30c

95 °C	WCS	3662 ± 27de	2755 ± 36b	907 ± 29f	4370 ± 21d	1615 ± 14d	7.23 ± 0.01b	71.35 ± 0.36abc	87.53 ± 0.13b
	WCS + F1	3586 ± 31e	2836 ± 44ab	750 ± 30g	5727 ± 120ab	2891 ± 76a	7.51 ± 0.04a	72.07 ± 0.56a	91.08 ± 0.37a
	WCS + F2	3972 ± 22b	2809 ± 11ab	1163 ± 24e	5542 ± 57b	2733 ± 65ab	7.06 ± 0.03c	70.52 ± 0.21bcd	85.63 ± 0.39c
120 °C	WCS	3736 ± 18cd	2892 ± 14a	844 ± 11fg	4453 ± 65cd	1561 ± 51d	7.26 ± 0.03b	70.37 ± 0.26bcd	87.97 ± 0.32b
	WCS + F1	3657 ± 57de	2841 ± 36ab	816 ± 28fg	5746 ± 45a	2904 ± 10a	7.55 ± 0.11a	71.42 ± 1.09ab	91.533 ± 1.28a
	WCS + F2	4062 ± 17ab	2086 ± 85de	1976 ± 87b	3768 ± 83f	1682 ± 167d	7.13 ± 0.02bc	70 ± 0.05cd	86.47 ± 0.21bc
140 °C	WCS	3809 ± 39c	2437 ± 13c	1372 ± 26d	4069 ± 30e	1632 ± 19d	7.26 ± 0.012b	70.43 ± 0.28bcd	88 ± 0.13b
	WCS + F1	3652 ± 67de	1978 ± 81de	1673 ± 40c	4590 ± 62c	2611 ± 27b	7.57 ± 0.07a	71.52 ± 0.36ab	91.83 ± 0.81a
	WCS + F2	4090 ± 36a	1697 ± 42e	2393 ± 36a	35 963 ± 42f	1900 ± 26c	7.09 ± 0.03c	69.85 ± 0.35d	85.92 ± 0.33c

120 °C	HACS	216 ± 2d	23 ± 4c	23 ± 4e	663 ± 14e	469 ± 15e	11.13 ± 0.10b	115.1 ± 0.07a	120.03 ± 0.06d
	HACS + F1	252 ± 6c	263 ± 6b	—	1341 ± 14b	1078 ± 17b	10.99 ± 0.012bc	115.12 ± 0.13a	120.1 ± 0d
	HACS + F2	246 ± 13^a^/355 ± 11a	333 ± 10a	23 ± 2d	1357 ± 13b	1105 ± 19b	11.61 ± 0.11a	88.18 ± 0.40^b^b	120.05 ± 0d
140 °C	HACS	227 ± 8d	—	218 ± 1c	739 ± 9d	735 ± 16d	10.73 ± 0.01d	115.08 ± 0.03a	129.73 ± 0.12c
	HACS + F1	276 ± 4b	12 ± 2d	264 ± 2b	890 ± 6c	878 ± 5c	10.95 ± 0.04c	115.25 ± 0.30a	132.28 ± 0.38a
	HACS + F2	234 ± 10^a^/345 ± 5a	10 ± 3e	335 ± 4a	1645 ± 49a	1635 ± 52a	10.887 ± 0.03cd	88.37 ± 0.16^b^b	131.45 ± 0.33b

a Both peaks in HACS gels are presented, even though the first peak is most likely a result of F2 addition, while the second peak most likely represents pasting behaviour of the starch gel.

b Pasting temperature corresponds to first peak in HACS-HX profiles at 120 and 140 °C.

CS, WCS, and HACS show distinctly different pasting curves. CS showed no changes in peak viscosity (1346, 1328, and 1333 cP, at 95, 120, and 140 °C, respectively), but it had a decreasing trend in trough viscosity (1158, 606, and 457 cP at 95, 120, and 140 °C, respectively). This indicates thermal degradation due to temperature increase. Trough viscosity is a measure of the lowest viscosity reached during holding stage. The starch granules collapse due to prolonged shearing and heat, therefore reducing viscosity. CS-120 had the highest final viscosity, followed by CS-95, and CS-140 (2393, 2190, and 1446 cP, respectively). The starch matrix formed at the end of a RVA measurement is dependent on leached polymers and swollen granules which retrograde and create starch gel.^37,41^ Polymer leaching increased with temperature, but at 140 °C, thermal degradation may have reduced network formation. In contrast, fewer polymers leached at 95 °C. This may explain why CS-120 exhibited the highest final and setback viscosity. Previously published works showed a decrease in final viscosity of conventional CS as the temperature increased (95 to 140 °C).^39,40^ However, the heating/cool rate in this work was 12.3 °C min^−1^, while in the published works the rate was 6 °C min^−1^. This could have impacted the starch gel properties, as it was shown that the rate affects the extent of starch granules swelling.^37^ There were no changes in pasting temperature of CS as pasting occurred at below 95 °C. Temperature at peak viscosity of CS-120 and CS-140 did not differ (100.88 and 101.30 °C, respectively), while CS-95 had a lower peak temperature (94.98 °C). This shows that CS had higher viscosity and, therefore, more swelling in high temperature RVA profiles (120 and 140 °C) due to increased polymer leaching.

WCS showed the highest peak viscosity when compared to CS and HACS. Waxy starches are known to have higher peak viscosity, more pronounced through viscosity, and lower setback than their conventional counterparts. This is due to the lack of amylose, which slows granule swelling and contributes to setback viscosity by reassociating into a matrix during cooling stage.^42^ Moreover, amylopectin (particularly short chained) can form hydrogen bonds with H_2_O during heating, thus causing starch granule swelling and increase in viscosity at lower temperatures than amylose-containing counterparts.^41^ WCS showed a slight increase in peak viscosity with increased temperature (WCS-95 = 3662 cP, WCS-120 = 3736 cP, WCS-140 = 3809 cP). At 140 °C, WCS had the lowest trough viscosity (2437 cP, as compared to 2775 cP at 95 °C and 2892 cP at 120 °C) and, consequently, the highest breakdown viscosity (1372 cP, as compared to 907 cP at 95 °C and 844 cP at 120 °C). This is reported in other works as well and is due to higher degree of disintegration of starch granules and, potentially, thermal deterioration.^40^ There were no changes in time of peak viscosity, pasting temperature, or temperature at peak viscosity, which suggest that WCS gelatinised below 95 °C. It has been reported that gelatinization temperature of WCS was 71 °C, as analysed by differential scanning calorimetry.^43^ Temperature influence is clearly seen in HACS pasting profile. At 95 °C, HACS did not gelatinise and its RVA profile shows low viscosity. Typical RVA curve with a defined peak viscosity can be seen in HACS-120 (216 cP) and HACS-140 (227 cP). This is expected, since high amylose starches have higher gelatinisation point, and increased temperature allows gelatinisation and granule swelling.^39,40^ High amylose starches overall have a lower viscosity and swelling properties than their conventional or waxy counterparts due to the lack of amylopectin, which is why at all temperatures, HACS shows lower viscosity than CS or WCS. Peak viscosity of HACS was previously reported (240 cP at 120 °C and 251 cP at 140 °C) and was similar to the results here.^44^ At 140 °C, there was a sharp decrease in trough viscosity, where it reached viscosity values so low their accurate determination was not possible. This can be seen in other works as well and can be attributed to thermal degradation.^40,44^ HACS-120 had a lower final viscosity than HACS-140 (663 and 739 cP, respectively), which is different from previous findings.^44^ However, our RVA profile ended at 25 °C, while in the referenced papers the RVA profile ends at 50 °C. At 50 °C (cooling stage), HACS-120 had a viscosity of 523 cP, while the viscosity of HACS-140 was 98 cP, agreeing with the final viscosity results in published literature.^44^ HACS-140 showed higher temperature at peak viscosity than HACS-120 (129.73 and 120.03 °C, respectively), concurring with the higher swelling and viscosity at higher temperatures.

(2) Influence of fibre addition on pasting characteristics of the 3 corn starch gels with varying amylose content

With fibre addition, we see major changes in pasting profiles (Fig. 2). The F1 slightly increased viscosity, while F2 had a more pronounced viscosity increase and it changed appearance of pasting curves, particularly at 120 and 140 °C. Appearance of a viscosity spike at ∼80 °C in the cooling stage can be seen in all starches, but it was the most prominent in HACS. It is likely that said change is due to strong gelling properties of F2. Since RVA is conventionally used to measure paste viscosity, rather than gel viscosity, it is possible that the RVA paddle partially breaks the solid gel, and the paddle continues to spin without accurately reporting viscosity. In RVA profile, this is seen as turbulent, uneven, and non-smooth curves. In this work, pasting curves show good reproducibility and mainly smooth lines. It was reported that psyllium husk powder changed the pasting of potato and pea starch gels, but no changes were seen for corn starch.^45^ However, the studied gels had higher concentrations of starch than ours (14 *vs.* 7.5%, respectively), and the whole psyllium was used, as opposed to fractions in our work. Another work studying corn starch replacement with whole psyllium showed RVA curve irregularities when the replacement level was 10%.^19^ Similarly to our work, Ren *et al.* reported appearance of an additional peak at 85 °C when psyllium powder was added to rice flour, suggesting amylose-psyllium interactions.^46^ It is possible that the interactions contribute to the emergence of this additional peak, however, when observing RVA profile of psyllium fractions without starches, the peak is evidently present in F2 (SI Fig. S1). It has been suggested that psyllium shows self-association at around 85 °C, temporarily binding and reducing the available water, which is supported by our results, as well.^46^ The F1 showed low viscosity at all temperatures (SI Fig. S2). This could be explained by galacturonic acid, which can increase solubility of *P. ovata* fibre.^15^

Fibre addition increases starch viscosity during pasting, particularly in starches with normal amylose content compared to waxy starches, as observed in our study. This happens due to synergistic interaction between fibres and leached amylose.^4,47^ At 95 °C, F1 increased peak viscosity by 14% in CS but had no effect on WCS, while F2 raised peak viscosity by 34% in CS and 8% in WCS (Fig. 2A and D). At high temperature treatments, WCS+F2-120 and WCS+F2-140 had higher peak viscosity than corresponding WCS gels (by 9 and 7%, respectively), but lower trough viscosity (by 28% and 30%, respectively) (Fig. 2E and F). The increase in peak viscosity could be due to WCS granules swelling at high temperature which then increases F2 concentration in the continuous phase, leading to higher viscosity. That is followed by the holding phase of the RVA profile in which the phase separation and thermal deterioration cause lower viscosity in WCS+F2 than in WCS, even though solids concentration is higher in WCS+F2 than WCS (7.87 and 7.5%, respectively). In HACS, there are clear differences when applying the two fractions – F1 increased overall viscosity, while it seems that F2 viscosity behaviour overwhelmed the pasting properties of HACS, especially at 120 and 140 °C (Fig. 2H and I). When F2 is added to HACS, a peak appears before the peak viscosity (minute 9, Fig. 2H and J). In HACS+F2-120, the viscosity of said peak is 246 cP, while in HACS+F2-140 it is 234 cP. This is, again, probably a result of F2 having stronger viscosifying effects than HACS, as this early peak is visible in RVA profile of F2 by itself (SI Fig. S2). So far, one published study reported on 3% addition of whole psyllium husk to corn starches with varying amylose contents.^25^ The sample preparation in the study included hydrothermal treatment of psyllium and starch, followed by drying, and the dried fibre-starch powder was then subjected to RVA analysis at 95 °C. Therefore, it cannot be directly comparable to our work. The authors reported no significant change to peak viscosity when psyllium was added to waxy and normal corn starch and increase in peak viscosity when added to high amylose corn starch.^25^ This partly agrees with our findings, as we report increase in peak viscosity when both psyllium fractions were added to CS and HACS, and for WCS we report increase only when F2 was added, but not F1. When soluble and insoluble fibre from corn bran was added to CS, contrasting trends were reported – soluble fibre decreased overall viscosity, while insoluble fibre caused an increase.^48^ Even though corn bran fibre is very different to *P. ovata* fibre, this work demonstrates that differently extracted fibre from the same source can cause varied results when added to starch gels. The authors described hydrogen bonding between the starch and soluble fibre, while insoluble fibre was embedded into the starch network.^48^ On the other hand, with fibre addition to different starches, the effect is dependent on specific starch-fibre combinations and their interactions. For example, incorporation of xanthan gum to CS, WCS, or HACS caused viscosity increase due to different mechanisms of interaction, as is seen in our work as well.^49^ In xanthan–CS, xanthan–amylose interactions increased peak viscosity. In HACS, xanthan raised viscosity as starch did not paste at 95 °C. In xanthan–WCS, phase separation concentrated xanthan in the continuous phase during granule swelling, increasing viscosity.^49^ Zhang *et al.* similarly found that phase separation likely contributed to increased viscosity when guar gum was added to WCS at 130 °C.^47^ Guar gum showed synergistic effects with HACS containing 50% amylose, while the impact was reduced in HACS with 70% amylose, indicating a dominant role of amylose in pasting behaviour.^49^ In our work, setback viscosity increased with F1 and F2 addition across all starches and treatments (Table 2). Corn bran arabinoxylans have been shown to increase setback viscosity in CS by creating a fibre network that concentrates leached amylose, promoting nucleation and crystal growth.^50^

Pasting temperature marks the onset of viscosity increase as granules swell and amylose begins to leach, initiating paste formation.^37^ F1 and F2 decreased pasting temperature of CS (by approximately 9 °C for both fibres). This is expected, as fibre addition increases viscosity, thus pasting will occur at lower temperature. In WCS, F1 slightly increased pasting temperature while F2 caused a slight decrease, though changes were modest (1–2 °C), further suggesting that amylose–fibre interactions predominantly influence pasting behaviour in CS. It has been proposed that amylose-fibre synergistic interaction are greater determinants of viscosity increase than phase separation.^47^ In HACS, F1 did not change pasting temperature. The early peak observed in HACS+F2 gels (minute 9, Fig. 2H and J) results in a significantly lower recorded pasting temperature (88 °C *vs.* 115 °C in HACS), though this may not represent a true change in the starch's pasting temperature. Altogether, it is evident that changes in pasting temperature are dependent on starch-fibre combination. F1 addition delayed peak viscosity, most notably in CS at 95 °C, where the peak shifted from 8.67 to 10.17 minutes. In contrast, F2 had little to no effect or slightly accelerated peak time. A similar pattern was observed in WCS, with F1 causing delays and F2 slight acceleration, though differences were smaller (20–30 seconds; Table 2). No consistent trend was observed in HACS.

To the best of our knowledge, no work has explored the addition of psyllium fractions in corn starch, however a recently published paper has shown the effect of psyllium with varying molecular weights in rice starch.^51^ All fractions decreased peak viscosity, trough viscosity and final viscosity, which is different than the pasting curves reported here. This was explained by limiting the gelatinisation of starch granules, and high solubility of psyllium fractions, which decreased the overall viscosity. However, it should be noted that the composition of psyllium fractions was described as predominantly glucose (>91%),^51^ which is quite different than composition of fractions used in this work, or in other published works, where xylose and arabinose were the main monosaccharides.^15,20,21^

Overall, RVA pasting profiles show that increased temperature treatments affected each starch differently. Fibre addition mostly led to higher viscosity, albeit there are clear differences between F1 and F2 effect on individual starch, and between starch-fibre combinations.

### 3.3. Techno-functional properties: texture analysis and freeze–thaw syneresis

**Fig. 3:**
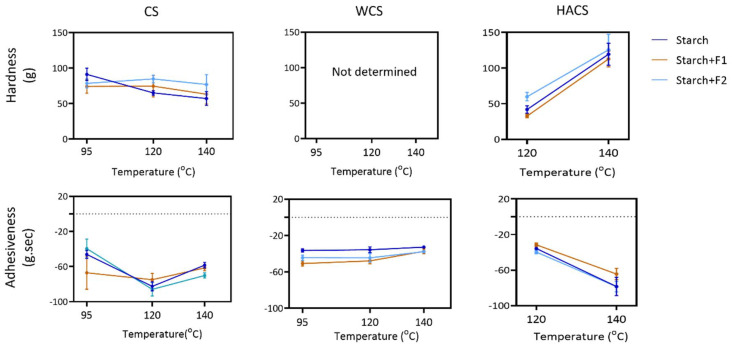
Texture analysis parameters (hardness and adhesiveness) of all gels at 3 temperature treatments. Dark blue colour represents starch gels, brown colour represents starch + F1, while light blue represents starch + F2 gel. Lines are presented to guide the eye.

Fig. 3 presents the texture profiles (hardness and adhesiveness) of starch and starch–fibre gels, excluding HACS at 95 °C and WCS, which did not form self-supporting gels suitable for hardness measurement. Fibre addition had minimal impact on texture, while temperature influenced HACS and CS hardness, with little effect on WCS. Increase in temperature negatively affected HACS, and gels treated at 140 °C showed significantly higher hardness.

Consistent with our findings, Tian *et al.* used high-temperature RVA (95–140 °C) on various starches and found increased hardness in two high-amylose maize starches, with no effect on other types.^40^ Similar trends can be seen in Liu *et al.*'s work – hardness of two high amylose starches increased as the temperature increased.^39^ High amylose starches are gelatinised by high temperature, which leads to granule swelling and amylose dispersion. Upon cooling amylose re-associates into denser, stronger matrix. Therefore, hardness of HACS at 140 °C is higher than at 120 °C (by 184.2%) (SI Table S1). There is a positive correlation between hardness and leached amylose (correlation coefficient = 0.85; leached amylose results are discussed in section 3.5.). This supports the hypothesis that greater amylose leaching, along with granule swelling and breakdown, leads to increased gel hardness.

While Liu *et al.* reported a slight decrease in WCS adhesiveness with temperature,^39^ our results showed no significant change. We observed a strong negative correlation between setback viscosity and adhesiveness (*r* = –0.74; SI Fig. S3B), consistent with the understanding that increased setback viscosity reflects greater amylose and amylopectin reassociation during cooling.^37^ Liu *et al.* also reported a significant decrease in hardness of normal maize starch at 140 °C due to thixotropic breakdown and thermal degradation, aligning with our findings for CS: 91.1 g at 95 °C, 65.1 g at 120 °C, and 57.1 g at 140 °C (SI Table S1).^39^

Effects of F1 and F2 on starch gel texture mostly did not differ from each other. Noticeable differences between HACS+F1 and HACS+F2 can be seen at 120 °C, where F1 resulted in lower hardness (by 83.2%). However, there was no difference between HACS+F1 and HACS+F2 at 140 °C, possibly due to thermal degradation of HACS at higher temperature as well as increased leached amylose (see section 3.5.), and decreased viscosity of the fibres at 140 °C (SI Fig. S2).

Different levels of corn starch replacement (2, 5, 10%) with psyllium was explored in gels treated at 95 °C.^19^ A 2% replacement increased hardness, while 10% decreased it, however, it is difficult to discern whether the changes are caused by addition of psyllium, decrease in starch quantity, or both. In our work, psyllium was added, rather than replaced, and it reduces hardness at 95 °C, but not significantly. It is known that products with psyllium require increased water content to maintain appearance and texture.^52^ However, the interaction of psyllium with amylose and amylopectin—and its impact on texture—remains to be investigated.

Freeze–thaw stability is a key indicator of retrogradation and storage stability in starch-based products. Results shown in Table 3 demonstrates no significant changes in CS and WCS, irrespective of temperature treatment or fibre addition. F1 has shown slight decreasing trend in syneresis when added to CS, which was proportional to increase in temperature (CS+F1 syneresis was 5.23, 4.14, and 3.69% at 95, 120, and 140 °C, respectively). However, this decrease was not significantly different from CS at corresponding temperature. On the other hand, HACS showed high syneresis after 15 days of freeze–thaw treatment, which is due to its high amylose content. Amylose is the primary driver of retrogradation, as it initiates short-term retrogradation.^53^ Fibres reduced syneresis in HACS gels. At 120 °C, HACS exhibited 11.93% syneresis, which was reduced by 41.24% with F1. At 140 °C, syneresis increased to 13.19%, but was reduced by 31.39% and 37.07% with F1 and F2, respectively. It was shown that WCS had 10× lower syneresis than two types of CS with conventional amylose content at the end of 90 day cold storage.^53^ Xanthan gum addition (0.02 and 0.05%) showed protective effect on the two CSs, while it increased syneresis when added to WCS. This indicates that xanthan gum could slow down short-term retrogradation by restricting mobility of the amylose molecules.^53^ Psyllium has been shown to significantly improve syneresis when added to food products. For example, when added to fruit jellies, psyllium completely reduced syneresis (from 11.69 g/100 g for control sample, to 0 g/100 g).^54^

**Table 3:** Freeze-thaw syneresis of starch and starch-fibre gels at 3 temperature treatments. Different letters correspond to significantly different results at *p* < 0.05

		Syneresis (%)
95 °C	CS	4.11 ± 0.86ab
	CS + F1	5.23 ± 0.17a
	CS + F2	4.78 ± 0.18ab
120 °C	CS	4.14 ± 0.12ab
	CS + F1	4.14 ± 0.05ab
	CS + F2	3.92 ± 0.24b
140 °C	CS	4.59 ± 0.36ab
	CS + F1	3.69 ± 0.21b
	CS + F2	4.75 ± 0.83ab

95 °C	WCS	4.76 ± 0.72ab
	WCS + F1	4.39 ± 0.49ab
	WCS + F2	4.73 ± 0.66ab
120 °C	WCS	3.97 ± 0.38b
	WCS + F1	4.04 ± 0.48b
	WCS + F2	4.46 ± 0.34ab
140 °C	WCS	5.63 ± 0.62a
	WCS + F1	4.43 ± 0.57ab
	WCS + F2	4.29 ± 0.31ab

120 °C	HACS	11.93 ± 0.68ab
	HACS + F1	7.01 ± 0.48c
	HACS + F2	11.81 ± 2.38ab
140 °C	HACS	13.19 ± 1.06a
	HACS + F1	9.05 ± 0.34bc
	HACS + F2	8.30 ± 0.12c

Overall, both fractions show limited effects on the hardness and adhesiveness of the tested starches. Moreover, CS and WCS were not affected by temperature treatment, unlike HACS. While the presented texture analysis (penetration test) did not show significant changes (SI Table S1), the fibre addition may have advantages in compression texture profile analysis. Freeze–thaw syneresis measurements have shown no changes in CS and WCS, whereas fibres had positive effect (meaning lower water separation) in HACS, which is desirable in food product development.

### 3.4. SEM

**Fig. 4:**
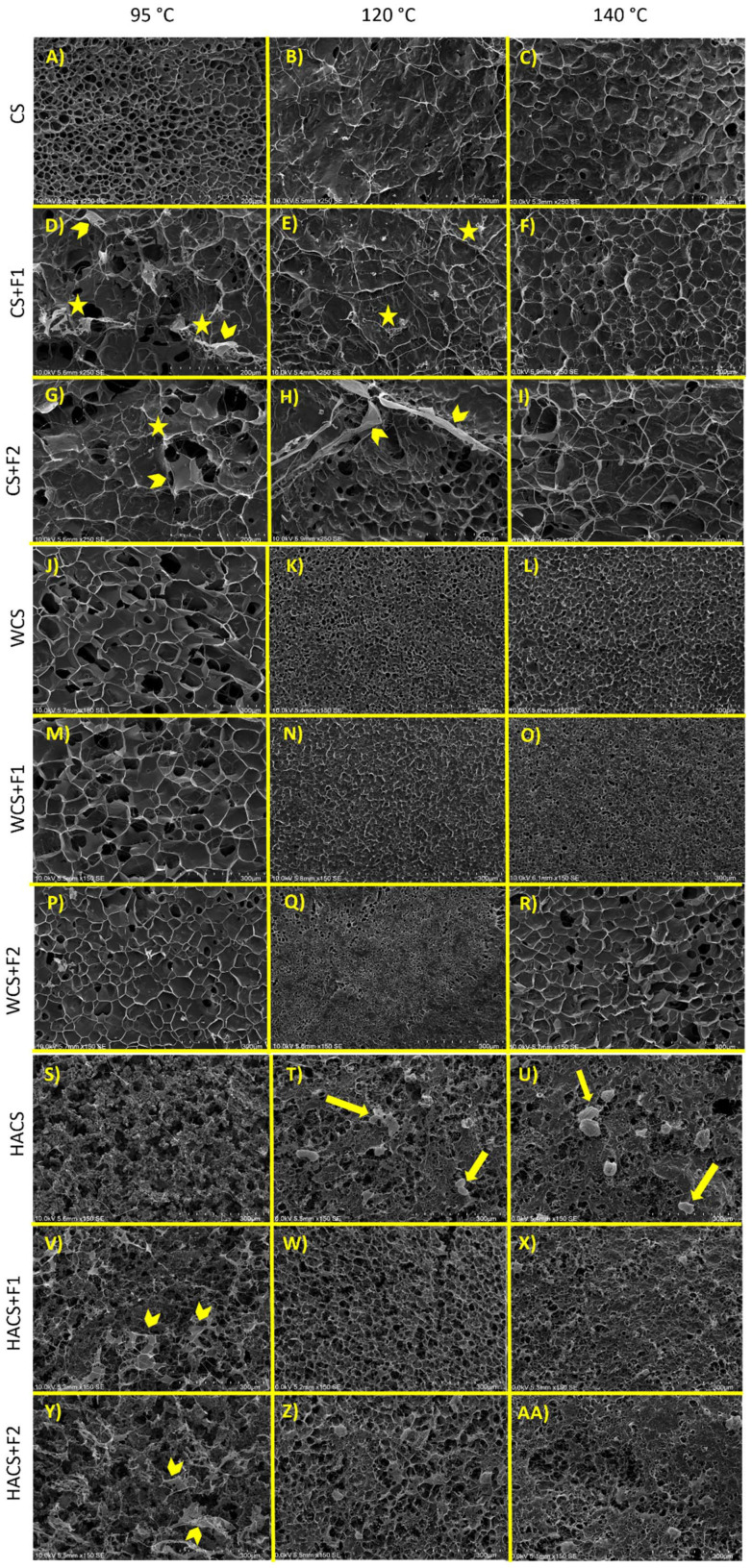
Scanning electron micrographs (SEM) of starch and starch-fibre gels. Temperature treatments specified at the top of the figure, and corresponding samples are written on the left-hand side. Stars point to fibre clusters, chevrons point to sheet-like fibre structures, and arrows point to ungelatinized starch granules.

Scanning electron microscopy (SEM) is oftentimes used for assessing starch gel structure. However, this technique has certain limitations. For conventional SEM, starch gels have to be dried, but the microstructure is changed by freezing and drying.^55^ Drying causes shrinkage, and we cannot adequately evaluate the distribution of starch and water (and fibre, if included). Visible pores are result of removal of ice crystals, rather than the result of starch structure itself and, lastly, we cannot differentiate between ordered and disordered parts of starch gel.^55^ Nevertheless, SEM can give us indication of differences in microstructure between samples, as the size, shape, and distribution of ice crystals formed during freezing are dependent on structural differences between starch and starch-fibre gels. SEM images of the gels explored in this work are presented in Fig. 4.

In CS-95, we can see uniform distribution of pores, whereas in CS-120 and CS-140 the structure appears flakier (Fig. 4A–C). This change in structure agrees with other published literature.^39^ In CS-120 and CS-140, spider-like filaments are visible, which is most likely leached amylose (Fig. 4B and C).^55^ Indeed, amylose leaching quantification (section 3.5.) showed increase with the increase of the temperature treatment. Moreover, the appearance of flaky structure rather than dense pores could be related with decrease in gel hardness, as reported in Fig. 3. The loss of porosity and development of a flaky structure suggest a weakening or loosening of the gel matrix, leading to reduced intermolecular interactions and diminished structural integrity, whereas the porous microstructure of CS-95 is typically associated with a more rigid and interconnected gel matrix, which contributes to higher gel hardness. In WCS, higher temperature treatments caused pronounced shrinkage of pores (Fig. 4J–L). SEM images show changes in formed ice crystals. WCS-95 shows ice crystals formation in the starch matrix (pore area of 4226 ± 1275 μm^2^), while in WCS-120 and WCS-140 interpenetration of water molecules in starch matrix supresses ice crystallization and the pores are smaller (319 ± 76 and 456 ± 86 μm^2^, respectively). At 95 °C, WCS structure (Fig. 4J) seems loosely packed, which is noted in Tian *et al.*'s work as well.^40^ When studying the effect of high temperature RVA on starch gel structure, they described conventional and waxy starches as having loosely packed and flaky structures, and high amylose starches as granular aggregates.^40^ In line with this, for HACS, it is evident that at 95 °C, it did not gelatinise as starch granules are easily visible (Fig. 4S–U). At 120 and 140 °C, the HACS matrix is compact, which indicates gelatinisation, with some swollen starch granules visible (Fig. 4T and U, marked with arrows).

The addition of F1 and F2 impacted structure of all three starches in different ways. In CS (Fig. 4D–J), both fibres form web-like matrix on the surface of starch, entanglements (marked with stars) and sheet-like areas (marked with chevrons). At 140 °C, both fibres show similar web-like matrix in CS, which points that the higher temperature combined with shear (160 rpm) might have dispersed the fibre, thus forming more uniform web fibre structure. The appearance of fibre webs and changes in the gel matrix appearance is most likely related to the macroscopic changes noted, such as viscosity. As reported, addition of both fibres led to increased peak viscosity of CS (Table 2). In WCS (Fig. 4M–R), the fibre ‘web’ is less visible, which is possibly due to amylopectin network of the starch predominating fibre network. Interestingly, in WCS+F2-120, a change in distribution of pores could indicate phase separation as, most likely, there is a change in size of ice crystals formed during freezing and subsequently dried, leaving pores at the surface (Fig. 4Q). Accordingly, it was previously mentioned (section 3.2.) that phase separation may be related to increase of peak viscosity and decrease of trough viscosity when fibres are added to WCS at 120 and 140 °C, thus again microscopic changes in gel structure could explain the changes seen in techno-functional properties. In WCS+F2-140 the pore size is larger than in WCS+F2-120 (1800 ± 321 and 109 ± 29 μm^2^, respectively). The pores are similar to WCS+F2-95 (3237 ± 1185 μm^2^), however they seem deeper, which could indicate structure deterioration by higher temperature. A similar observation can be seen in Liu *et al.*'s work, where waxy maize starch heated at 140 °C had deeper, larger, and more elongated pores.^39^ In HACS, sheet-like fibre structures can be seen for both F1 and F2 at 95 °C (Fig. 4V and Y, marked with chevrons), and at higher temperature treatments, a dense fibre matrix is apparent (Fig. 4W, X, Z and AA). It seems that addition of F1 could have led to increased gelatinization since there are less ungelatinized starch granules, whereas in HACS+F2 at 120 and 140 °C more ungelatinized starch granules are visible. When whole psyllium husk was added to wheat starch and treated at 90 °C for 10 min, it showed an irregular and rough surface.^56^ The surface appears different from what we observed, however there were clear differences in starch-fibre gels: use of whole *vs.* fractionated psyllium, ratio of psyllium to starch (1 : 1 in the referenced paper), and sample preparation. Zhang *et al.* have studied the addition of guar gum and xanthan gum in two types of HACS and in WCS heated up to 130 °C.^47^ Their SEM images show that guar gum may promote amylose aggregation, and xanthan gum forms denser structure in HACS, but neither of added gums had significantly changed WCS structure. Our images show different trend, where both F1 and F2 did change WCS structure at 140 °C. However, psyllium (and the fractions used in this work) is different from guar gum and xanthan gum, so difference in WCS-fibre structure is not surprising.

### 3.5. Leached amylose

**Fig. 5:**
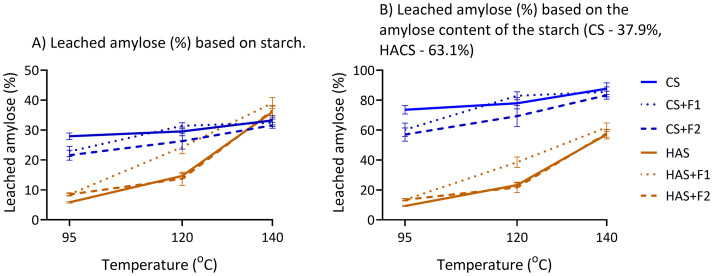
Amylose leaching in CS with and without fibre addition, and amylose leaching in HACS with and without fibre addition calculated: (A) based on starch content, (B) based on amylose content of individual starch (CS −37.9%, HACS −63.1%). Lines are drawn to guide the eye. There was no leached amylose detected in WCS.

Leached amylose refers to the portion of amylose that diffuses from swollen starch granules into the continuous phase during heating (above gelatinization temperature) and shear processing of the starch paste.^57^Fig. 5 shows leached amylose (%) of CS and HACS relative to total starch (Fig. 5A) and relative to the amylose content of the individual starch (Fig. 5B). There was no detected leached amylose for WCS (not shown). The exact values are shown in SI Table S2.

Higher temperature increased leached amylose in both starches, while fibre addition showed different effects. In CS, addition of F1 significantly decreased amylose leaching only at 95 °C (by 22.56%). F2 significantly decreased amylose leaching at 95 °C and 120 °C (by 18.19% and 10.99%, respectively). In HACS, the addition of F1 significantly *increased* leached amylose at 120 °C and 140 °C (by 65.44% and 8.30%, respectively), and addition of F2 did not significantly change amylose leaching. As HACS did not reach gelatinization at 95 °C, there was no significant difference between the samples. It is also evident that the majority of amylose content of CS leached out of starch granules at all temperatures (73.66% and 87.67% of amylose was leached at 95 °C and 140 °C, respectively), while for HACS only at 140 °C resulted in relatively high amylose leaching (57.12%).

Temperature treatment and heating rate have a pronounced effect on the amount of leached amylose.^57^ Considering that the rate of heating was kept constant in our experiments (section 2.3.), it can be concluded that the increased temperature has resulted in increased leached amylose in CS and HACS, which agrees with literature.^57^ However, the requirement for higher temperatures to gelatinize HACS, combined with the limitations of conventional methods (*e.g.*, RVA at 95 °C or hydrothermal treatment below 100 °C), has contributed to the limited literature on amylose leaching in HACS. Moreover, to the best of our knowledge, this is the first report on amylose leaching impacted by psyllium fibre. When wheat AXs (0.5%) of varying molecular weight were added to wheat starch, lower *M*_w_ AXs (up to 373 kDa) had no effect on amylose leaching, whereas higher *M*_w_ AXs (421, 482, and 754 kDa) reduced leaching.^11^ This was attributed to their restriction of gelatinization by limiting water availability. Similarly, corn fibre gum (0.5%) of varying *M*_w_ (186, 290, and 338 kDa) reduced amylose leaching in wheat and corn starch, with greater effect seen in corn starch.^10^ Adsorption of corn fibre gum onto the starch granule surface and a possible interaction with amylose were proposed as explanations, which could be at play in our CS-F2 system.

As mentioned, HACS+F1 has shown a contrasting effect on amylose leaching – F1 significantly *increased* the amount of leached amylose at 120 and 140 °C, with the most apparent increase at 120 °C (60.44% increase from HACS). F1 was characterised as low branched heteroxylan (A : X = 0.17) with minor pectin component (9.6%) (Fig. 1C). Conversely, densely branched wheat AX (A : X = 0.79–0.97) has been shown to have a lowering effect on amylose leaching in wheat starch.^58^ Pectin has been shown to decrease amylose leaching in wheat, potato, and corn starch.^9,59,60^ Therefore, the increase in amylose leaching is more likely driven by the low-branched heteroxylan in F1 than by its pectin content.

### 3.6. Starch hydrolysis by α-amylase and estimated glycaemic index (eGI)

**Fig. 6:**
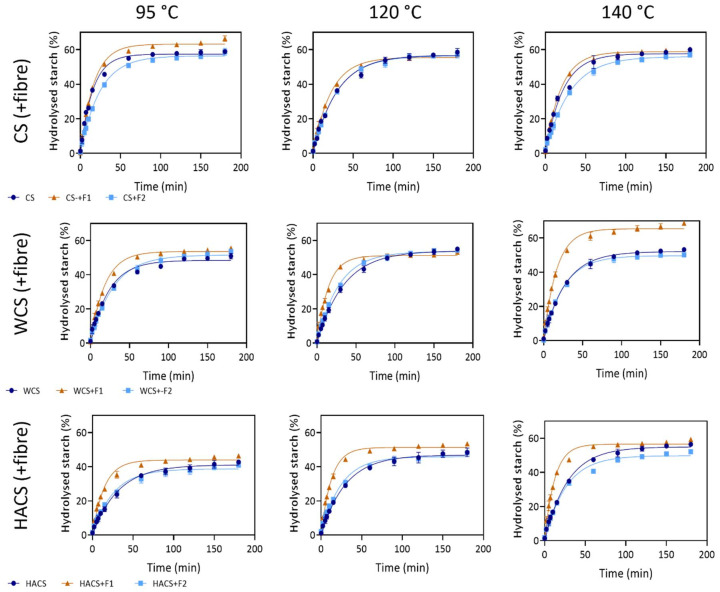
Starch hydrolysis curves of starch and starch-fibre gels (A) corn starch, (B) waxy corn starch, (C) high amylose corn starch). Dark blue line represents starch, brown line represents starch + F1, and light blue line represents starch + F2.

Starch hydrolysis by α-amylase is a useful predictor of metabolic response, as hydrolysis rate (constant *k*) and extent of hydrolysis (*C*_∞_) of a starch are clearly affected by fibre addition and temperature.^30^ Psyllium husk is known as a modulator of glycaemic response and has positive effect on glycaemic control, however studies of psyllium and its fractions in enzymatic starch hydrolysis are sparse. Here we present, to our knowledge, the first results of enzymatic hydrolysis of corn starch with varying amylose and added psyllium fractions. In Fig. 6, we show non-linear fitting model of hydrolysed starch and starch-fibre gels, and the associated hydrolysis parameters are presented in Table 4.

Starch hydrolysis parameters (measured and estimated extent (%) and rate of hydrolysis (*k*)) and goodness of fit (*R*^2^). Different letters correspond to significantly different results at *p* < 0.05

**Table:** 

(A)	CS-95	CS + F1-95	CS + F2-95	CS-120	CS + F1-120	CS + F2-120	CS-140	CS + F1-140	CS + F2-140
Measured extent of hydrolysis (%)	58.89 ± 1.32b	66.39 ± 1.63a	58.76 ± 1.99b	58.52 ± 2.13b	57.17 ± 0.71b	57.37 ± 2.23b	60.01 ± 1.07b	60.34 ± 0.32b	57.34 ± 1.70b
Estimated extent of hydrolysis (%)	57.39 ± 1.12bc	63.20 ± 0.44a	56.38 ± 1.14bc	56.80 ± 1.82bc	55.50 ± 0.27c	56.45 ± 1.35bc	57.68 ± 0.52bc	58.82 ± 0.50b	55.98 ± 1.06bc
Constant *k*	0.065 ± 0.0a	0.061 ± 0.004a	0.042 ± 0.001c	0.033 ± 0.001d	0.045 ± 0.004bc	0.034 ± 0.002d	0.046 ± 0.003bc	0.052 ± 0.003b	0.033 ± 0.002d
*R* ^2^	0.9917	0.9945	0.9950	0.9910	0.9922	0.9945	0.9850	0.9956	0.9940

**Table:** 

(B)	WCS-95	WCS + F1-95	WCS + F2-95	WCS-120	WCS + F1-120	WC + F2-120	WCS-140	WCS + F1-140	WCS + F2-140
Measured extent of hydrolysis (%)	50.81 ± 1.63cd	55.50 ± 0.59b	53.58 ± 1.00b	55.00 ± 0.69b	53.14 ± 0.87bc	54.26 ± 0.46b	53.14 ± 0.94bc	68.60 ± 0.67a	50.12 ± 1.06d
Estimated extent of hydrolysis (%)	48.38 ± 1.08e	53.56 ± 0.29bc	51.54 ± 1.24bcd	53.97 ± 0.45b	51.14 ± 0.80cd	53.49 ± 0.65bc	52.09 ± 0.70bcd	65.40 ± 1.47a	49.63 ± 1.10de
Constant *k*	0.043 ± 0.004c	0.055 ± 0.005b	0.035 ± 0.003cd	0.029 ± 0.002d	0.070 ± 0.004a	0.036 ± 0.003cd	0.036 ± 0.004cd	0.060 ± 0.005ab	0.040 ± 0.003cd
*R* ^2^	0.9826	0.9902	0.9871	0.9954	0.9875	0.9953	0.9937	0.9910	0.9937

**Table:** 

(C)	HACS-95	HACS + F1-95	HACS + F2-95	HACS-120	HACS + F1-120	HACS + F2-120	HACS-140	HACS + F1-140	HACS + F2-140
Measured extent of hydrolysis (%)	42.76 ± 0.94ef	46.32 ± 0.82f	40.99 ± 1.28de	48.38 ± 2.52d	53.39 ± 0.62bc	48.10 ± 1.29d	56.44 ± 0.97ab	59.22 ± 1.14a	52.05 ± 0.58c
Estimated extent of hydrolysis (%)	41.09 ± 1.27ef	43.90 ± 0.75de	38.69 ± 1.60f	46.85 ± 2.34cd	51.26 ± 0.60b	45.85 ± 1.03d	54.96 ± 0.90a	56.51 ± 0.19a	49.83 ± 0.07bc
Constant *k*	0.032 ± 0.003c	0.069 ± 0.006b	0.039 ± 0.003c	0.033c	0.079 ± 0.005ab	0.042 ± 0.003c	0.035 ± 0.002c	0.082 ± 0.009a	0.038 ± 0.004c
*R* ^2^	0.9850	0.9807	0.9791	0.9884	0.9898	0.9836	0.9921	0.9855	0.9863

When observing CS gels, the temperature treatment did not change the hydrolysis extent, while constant *k* was highest at 95 °C treatment, followed by 140 °C, and lastly 120 °C (0.065, 0.046, 0.033, respectively). The addition of F1 increased hydrolysis extent at 95 °C (by 12.7%), and it increased the hydrolysis rate at 120 °C (by 36%). There were no significant changes between CS and CS+F1 starch hydrolysis at other conditions. As for the addition of F2, decreases in constant *k* were found in samples treated at 95 and 140 °C (by 35.4 and 28.3%, respectively), though F2 did not change the extent of hydrolysis at any temperature. Overall, the most similar starch hydrolysis profiles between CS and CS with fibres can be noticed at *T*_max_ = 120 °C (Fig. 6A).

Hydrolysis extent in WCS was the lowest at 95 °C (48.38%), and it did not differ significantly between 120 and 140 °C treatments (53.97 and 52.09%, respectively). The lowest constant *k* was measured in 120 °C treatment (0.029). The addition of F1 significantly increased constant *k* at all temperatures, with the highest increase at 120 °C (141.4% increase). The highest change in hydrolysis extent can be seen at 140 °C, where addition of F1 resulted in a 29.1% increase. The F2 did not significantly change constant *k*, and it had an inconsistent effect on hydrolysis extent (increased at 95 °C by 9.2%, and no change at 120 and 140 °C) (Fig. 6B).

High amylose starches require higher temperatures to achieve gelatinization,^61^ therefore utilising HT-RVA gives us useful insight into hydrolysis behaviour of HACS with and without fibre. As expected, with increased temperature, both rate and extent of hydrolysis increased (Fig. 6C). F2 did not significantly change hydrolysis rate of HACS at any condition, and it decreased hydrolysis extent only at 140 °C (by 7.8%). Contrarily, F1 showed high hydrolysis rate increase (compared to HACS, F1 increased constant *k* by 115.6%, 139.4%, and 134.3% at 95, 120, and 140 °C, respectively). Moreover, F1 caused an increase of the hydrolysis extent at 120 °C (by 8.3%), while it had no effect at 95 and 140 °C.

Generally, F1 changed starch hydrolysis patterns by increasing hydrolysis rate in almost all cases (except CS-95 and CS+F1-95, where there was no significant difference), with the biggest changes in HACS gels. F2 had inconsistent effects: it mostly did not significantly change hydrolysis rate, except in CS+F2-95 and CS+F2-140, where it showed decrease compared to CS gels. As for hydrolysis extent, addition of F1 either increased or did not change hydrolysis extent, and F2 either decreased or did not have an effect.

Corn is one of the most widely consumed crops globally, and efforts to modify its digestibility aim to reduce its glycaemic index.^62^ In literature, we can find reports of additions of different fibres to corn starch. Some researchers report methodology similar to ours with results showing *k* and *C*_∞_, while others utilise Englyst assay which reports rapidly digested starch (RDS), slowly digestible (SDS) and resistant starch (RS).^63^ Even though we cannot directly compare those results to ours, we can cautiously juxtapose trends in RDS and RS with trends in hydrolysis rate and extent – starches with high hydrolysis rate and RDS can lead to spike in blood glucose level, and starches with high RS and low hydrolysis rate and extent can improve glycaemic control.^63^

At 95 °C treatment, CS showed the highest hydrolysis extent and rate, followed by WCS, then HACS. This agrees with work published by Zheng *et al.* who assessed *in vitro* sequential dynamic digestion of CS, WCS, and HACS gels treated at 95 °C for 30 min.^64^ In the intestinal stage, hydrolysis of gastric digesta (180 min) of the three starches followed the same trends as in our results (CS > WCS > HACS). They explained that HACS gel contained native starch granules which are less susceptible to enzymatic attack, while highly viscous WCS was more difficult to hydrolyse by α-amylase. Liu *et al.* reported that resistant maize starch (59.1% amylose) treated at 120 °C showed the highest digestibility when compared to treatments at 95 and 140 °C (which did not differ from each other).^44^ Their explanation was that at 120 °C, starch granules were not dispersed enough (as shown by SEM) to develop enzymatically resistant structures during cooling. That said, our results do not agree with theirs, as we have shown the expected increase in the hydrolysis rate and extent with the temperature increase.

Psyllium husk is proven to be beneficial in glycaemic control. When added to wheat, potato, and tapioca starch, psyllium lowered RDS and increased SDS and RS in all three starches.^18^ This can be attributed to increased viscosity caused by psyllium and by reducing the amount of water available for gelatinization. However, psyllium addition was 50%, which is quite high and not feasible in food product formulation. Addition of 3% psyllium in corn starches with varying amylose contents which were hydrothermally treated and annealed showed increase in SDS and RS in all starch types.^25^ However, this work employed vastly different sample preparation than ours, so it cannot be directly compared. In food products, psyllium showed decrease in starch digestibility of fibre enriched white bread: when compared to xanthan gum and lambda-carrageenan, 3% psyllium addition showed the lowest glycaemic index (61, 54, and 46, respectively).^65^ Santamaria *et al.* developed a sequential RVA protocol with the addition of α-amylase where they studied the relationship between changes in viscosity caused by enzymatic hydrolysis of starch.^45^ They reported an increase in hydrolysis rate *k* when psyllium was added to CS, which is an opposite trend from the previously mentioned studies. In their work, CS had a hydrolysis rate of 0.311 min^−1^, and when 0.5% and 2.5% psyllium were added, *k* increased to 0.484 and 0.595 min^−1^, respectively.^45^ It is clear that there is research interest in the effect of psyllium on starch digestibility, but there is less information about how psyllium fractions influence patterns of starch hydrolysis.

Numerous studies have investigated the impact of fibre addition on starch hydrolysis, with most reporting a beneficial effect—namely, reduced starch digestibility.^66^ Increasing concentrations of pectin (0.5–10%) added to corn starch resulted in a proportional decrease in RDS and an increase in SDS and RS.^67^ Corn bran arabinoxylans of varying crosslinking degree decreased hydrolysis rate and extent of corn starch, and highly crosslinked arabinoxylan (H-CLAX) caused the lowest *k* and *C*_∞_.^50^ This was explained by high final viscosity which could have inhibited the diffusion of α-amylase. However, changes in hydrolysis rate and extent are often dependent on the physico-chemical properties of fibre or gum which is added to the starch gel (such as molecular weight, branching, charge, and concentration). On the other hand, some papers report increases in starch hydrolysis susceptibility, which is apparent in our work when F1 was added. For instance, when pectin, guar gum, CMC, xanthan, and HPMC were added to corn starch at concentrations of 1–4%, only HPMC at 3% and 4% reduced the hydrolysis rate, while all other gels showed a significant increase.^68^ The extent of hydrolysis was not consistently affected by gum type or concentration. The authors suggested that gum–amylose interactions might inhibit retrogradation and enhance enzymatic susceptibility, while increased viscosity could hinder α-amylase activity. These mixed effects highlight the need to evaluate each gum–starch combination individually, as no universal trends were observed.^68^ In another work, addition of xanthan and konjac gum (0.05–0.3%) to mung bean resistant starch led to increased digestible starch, and decreased resistant starch.^69^ This indicates different digestion patterns when gums are added to resistant starches (as opposed to conventional starches). This concept should be further explored.

In our study, the mechanisms influencing starch hydrolysis vary based on both the starch and the fibre. Notably, the significant increase in hydrolysis rate with F1 addition warrants further examination. Next, we discuss potential mechanisms.

F1 addition did not increase viscosity as markedly as F2, and in WCS, it even reduced peak viscosity (Fig. 2). F1 also had a lower hydrated volume than F2, with water swelling capacities of 5.27 mL g^−1^ and 9.67 mL g^−1^, respectively. This suggests that F1 retains less water, resulting in more interstitial (free) water rather than water bound within a viscous fibre matrix. Consequently, α-amylase may be concentrated in these low-viscosity regions, enhancing its accessibility to starch and accelerating hydrolysis. This concept has been proposed in works exploring pectin addition to starch, however this hypothesis has not been definitely proven.^12^ More precisely, this possibility could be true for some fibres with specific intrinsic properties, or only for certain concentrations. It may be that this “water distribution” theory might explain results seen in WCS+F1 starch hydrolysis. When observing SEM Images of WCS, WCS+F1 and WCS+F2 at 140 °C, there is apparent difference in pore size, which is attributed to ice crystals formation (Fig. 4M–O). WCS+F1-140 has smaller pore size than WCS-140 and WCS+F2-140 (173 ± 45, 456 ± 86, and 1800 ± 321 μm^2^, respectively) and less scaly surface, which could represent interpenetration of water into the matrix (interstitial water phase), and suppression of ice crystals formed when storing the gels at −80 °C. As mentioned, interstitial water could lead to increased relative concentration of amylose, which would lead to increased starch hydrolysis rate and extent (as reported in Table 4).

Another possible explanation for the F1-induced increase in starch hydrolysis involves its impact on amylose leaching. It has been shown that higher temperature leads to increased amylose leaching accompanied by amylopectin leaching as well.^57^ We report agreeing findings as well (Fig. 5). We speculate that F1 could have disrupted amylose network, either by (1) dilution with interstitial water which would lead increased amylose leaching, (2) interfering with amylose network and disrupting amylose-amylose interactions, or (3) interacting with amylose itself which could make amylose chains more susceptible to enzymatic attack. This would explain the increased constant *k* and hydrolysis rate in HACS at all temperatures. It is possible that this could also explain increased hydrolysis rate and extent in WCS+F1-140 where perhaps even non-measurable amylose (which would have been leached the most at 140 °C) could have caused faster enzymatic attack. Additionally, leaching of low molecular weight amylopectin could have also been increased by temperature and F1, which could explain why F1 seemed to increase both starch hydrolysis parameters in WCS. However, to further elaborate and prove this theory, analysis like size exclusion chromatography would be advisable.

Contrarily, F2 does have higher viscosity, which could have slowed down enzymatic mobility. The F2 can act as “shield” around starch, thus making it inaccessible to α-amylase, which is often proposed for highly viscous fibres.^18^ Due to its high water absorption, F2 could also limit the amount of water available for starch gelatinisation, thus leading to decreased susceptibility to enzymatic hydrolysis. This could be the case for HACS, as the SEM images show the ungelatinized starch granules when F2 is added, and no granules or smaller amount when F1 is added (Fig. 4W, X, Z and AA). It has been proven that both physico-chemical properties and viscosity has significant effect on starch hydrolysis – when 4 different soluble fibres (xanthan gum, konjac glucomannan, hull-less barley β-glucan, tamarind seed gum) were added to corn starch at the same initial viscosity level, they showed similar results of starch hydrolysis extent, but significant differences in the digestion rate constant.^70^ However, whether these possible mechanisms can explain the contrasting effects of F1 and F2 seen here is highly speculative and would require further analyses to show not only the interactions between starches and fibre fractions, but also if the fibre fractions have any interaction with α-amylase.

Glycaemic index (GI) is a measurement of carbohydrate-containing foods and their glycaemic response *in vivo*. GI has been correlated with metabolic health and is dependent on the rate end extent of starch hydrolysis.^30^ Our eGI calculations (estimated GI) show that F1 increased eGI of CS-95, WCS-95, WCS-140, and in all HACS gels (SI Fig. S4). The F2 decreased eGI in CS-95 and HACS-140. Unsurprisingly, eGI was significantly positively correlated with the hydrolysis extent for all 3 starches (CS = 0.91, WCS = 0.96, HACS = 0.97), and with hydrolysis rate for CS (0.89) and HACS (0.68) (SI Fig. S3). For WCS, correlation between hydrolysis rate and eGI was positive, but not significant (0.62). Considering that eGI is an indicator of glycaemic response, F1 could lead to its increase, which would not be advisable for people who have indication of metabolic syndrome, but might be applicable for developing rapid-energy-supply products for athletes and during high intensity activity, or supplements for malnutrition which, besides rapidly digested starch would have potentially prebiotic fibre addition.^71^ However, use of F2 would be advisable when developing further starch-based products for people with metabolic disorders or in products that are known to have high GI, such as gluten-free bread.^72^

## 4. Conclusions

To date, no previous studies reported interaction between starch type, temperature treatment and fibre fractions of *P. ovata* husk. Most of the studies have focused on the effect of high temperature on different starches, while the combined effect of fibre and temperature is underexplored. In light of the ongoing efforts to enhance dietary fibre intake and to nutritionally optimize starch-based products, we propose that our research will address the existing knowledge gap regarding the properties and behaviour of starch-fibre gels subjected to high-temperature treatments. This work explored the addition of two fibre fractions (F1 and F2) from *P. ovata* husk in 3 starches with varying amylose content at three different temperatures (95, 120, and 140 °C). The two fractions were heteroxylans with varying pectin content and arabinose to xylose ratio (F1 had 9.6% pectin and A : X = 0.17, while F2 had no pectin and had A : X = 0.36). F2 was more viscous and had higher hydrating capacities. F1 and F2 had contrasting effect on starches, which also depended on the starch type, and changed the functionality of starch-fibre gels. F1 slightly increased viscosity of the starches, while F2 changed the appearance of the RVA curve causing several new peaks. The higher temperature caused thermal degradation in all starch types. Both fibre and temperature had limited effects on texture properties and freeze–thaw syneresis. The F1 significantly increased starch hydrolysis rate, which was explained by increase in leached amylose that could have been hydrolysed faster, while F2 had no effect, or it slightly decreased starch hydrolysis parameters.

This work included a wide range of starch and starch-fibre gels with focus on analyses which would be of use in food and health industry. For example, highly viscous fibre (F2) could be used for formulation of products with lower glycaemic index, while soluble fibre with low viscosity (F1) could be used for starch-based products where fast glucose release is desirable. In addition, for fabricating fibre-enriched products where the requirement is that texture and viscosity during thermal treatments remain similar to the original product, then F1 would be advisable. However, mechanisms that drive the reported results should be further explored. We propose that the use of FTIR to assess changes in chemical bonds and conformation, detailed rheology studies, cryo-SEM and size exclusion chromatography of leached polymers could provide insights into interactions between starches and fibres and the gels’ structure. Moreover, using confocal microscopy with fluorescent labelling could explain more detailed differences in structure. Furthermore, the sequence of starch–gel preparation is an influential factor. We propose that investigating alternative mixing strategies, such as dissolving the fibre prior to starch incorporation, may provide new opportunities to optimize starch–fibre gel formation and ultimately improve starch-based food products.

Finally, novelty and significance of this paper lie in studying *P. ovata* fractions, rather than whole husk, in starch food models. We included high amylose corn starch for which, to the best of our knowledge, there are no reports on amylose leaching at high temperature (>100 °C). Furthermore, studies about fibre effect on high amylose starches are sparse. F1 fibre used in this work is heteroxylan with a low A : X ratio (0.17), for which there are also limited studies. To conclude, this work clearly shows that fibre fractions extracted from the same source can have contrasting effect in starch-based gels, which should be taken into consideration when developing new food products.

## Author contributions

Conceptualization – L. Š., J. M. C.; formal analysis – L. Š.; funding acquisition – R. A. B., G. E. Y.; investigation – L. Š., J. M. C.; methodology – L. Š., J. M. C.; supervision – R. A. B., G. E. Y., J. M. C.; writing – original draft preparation – L. Š.; writing – review and editing – L. Š., R. A. B., G. E. Y., J. M. C.

## Conflicts of interest

There are no conflicts to declare.

## Supplementary Material

FO-016-D5FO02366A-s001

## Data Availability

The data supporting this article have been included as part of the supplementary information (SI). Supplementary information is available. See DOI: https://doi.org/10.1039/d5fo02366a. Raw data can be made available upon request at corresponding author's email address.
