# Supplementary material for: Contrasting impacts of Plantago ovata fibre fractions on corn starch structure and digestibility
Source: Food Funct. 2025 Oct 27;16(22):8890–910. doi: 10.1039/d5fo02366a (PMC12581016; doi:10.1039/d5fo02366a)
Supplement: Supplementary file 1 [file FO-016-D5FO02366A-s001.zip › supplementary figures and tables.odt]

SUPPLEMENTARY FIGURES AND TABLES

Supplementary figure 1. Graphical diagram of RVA protocols.

A black screen with blue arrows

AI-generated content may be incorrect.

Supplementary figure 2. Pasting profile of F1 (a) and F2 (b) at 3 temperature profiles. Blue colour represents the pasting curve at 95 °C, green at 120 °C, and purple at 140 °C.

A screenshot of a graph

AI-generated content may be incorrect.

Supplementary figure 3. Correlation matrix for starch gels: (a) CS, (b) WCS, and (c) HACS. Green cells represent positive correlation, and red cells represent negative correlation. Bold values represent significant correlation coefficient.

A row of graphs with different colored squares

AI-generated content may be incorrect.

Supplementary figure 4. Estimated glycaemic indices (GI) for all starch and starch-fibre gels.

A group of blue and orange bars

AI-generated content may be incorrect.

Supplementary table 1. Texture parameters (hardness and adhesiveness) values presented as means of three replicates ± standard deviation. Different letters correspond to significantly different results at p<0.05.

|  |  | Hardness (g) | Adhesiveness (g/s) |
| --- | --- | --- | --- |
| 95 °C | CS | 91.1±8.7a | -46.1±4.5ab |
| CS+F1 | 74.2±9.5abc | -67.0±18.8cde |
| CS+F2 | 78.6±ab | -39.6±10.8a |
| 120 °C | CS | 65.1±3.1bc | -82.5±5.2ef |
| CS+F1 | 74.6±15.2abc | -75.0±7.2def |
| CS+F2 | 84.8±5.4a | -85.9±7.6f |
| 140 °C | CS | 57.1±9.9c | -58.4±3.3bc |
| CS+F1 | 63.3±13.7bc | -61.5±3.3bcd |
| CS+F2 | 76.9±14.0ab | -70.2±2.9cdef |
| 95 °C | WCS | - | -36.4±1.8a |
| WCS+F1 | - | -50.8±2.6c |
| WCS+F2 | - | -44.5±2.9b |
| 120 °C | WCS | - | -35.6±3.0a |
| WCS+F1 | - | -47.9±3.0bc |
| WCS+F2 | - | -44.5±3.0bc |
| 140 °C | WCS | - | -32.8±1.5a |
| WCS+F1 | - | -37.8±2.7a |
| WCS+F2 | - | -37.8±1.7a |
| 120 °C | HACS | 42.0±5.4bc | -35.7±0.9a |
| HACS+F1 | 32.7±2.6c | -31.5±1.9a |
| HACS+F2 | 59.9±6.0b | -39.8±2.0a |
| 140 °C | HACS | 119.4±15.4a | -78.5±5.8c |
| HACS+F1 | 112.8±11.1a | -64.4±6.3b |
| HACS+F2 | 125.7±22.0a | -78.7±5.8c |

Supplementary table 2. Amylose leaching in CS with and without fibre addition, and amylose leaching in HACS with and without fibre addition calculated: A) based on starch content, B) based on amylose content of individual starch (CS – 37.9%, HACS – 63.1%). There was no leached amylose detected in WCS. Different letters correspond to significantly different results at p<0.05.

| Leached amylose (%) based on starch content. | B) Leached amylose (%) based on amylose content of each starch (CS – 37.9%, HACS – 63.1%). |
| --- | --- |
| 95 °C | CS | 27.92±1.09 cd | HACS | 5.85±0.24 e | 95 °C | CS | 73.66±2.88 cd | HACS | 9.27±0 e |
| CS+F1 | 21.62±1.68 e | HACS+F1 | 8.48±0.34 e | CS+F1 | 60.26±4.45 e | HACS+F1 | 13.49±0.89 e |
| CS+F2 | 22.84±1.69 e | HACS+F2 | 8.51±0.56 e | CS+F2 | 57.07±4.43 bc | HACS+F2 | 13.44±0.53 e |
| 120 °C | CS | 29.56±1.45 bc | HACS | 14.70±0.89 d | 120 °C | CS | 78±3.82 bc | HACS | 23.29±1.41 d |
| CS+F1 | 31.43±1.02 ab | HACS+F1 | 24.32±2.21 c | CS+F1 | 82.94±2.69 ab | HACS+F1 | 38.54±3.50 c |
| CS+F2 | 26.31±2.68 d | HACS+F2 | 13.72±2.22 d | CS+F2 | 69.41±7.07 d | HACS+F2 | 21.74±3.51 d |
| 140 °C | CS | 33.23±1.50 a | HACS | 36.04±1.92 b | 140 °C | CS | 87.67±3.96 a | HACS | 57.12±3.05 b |
| CS+F1 | 32.43±1.26 ab | HACS+F1 | 39.03±1.88 a | CS+F1 | 85.57±3.33 ab | HACS+F1 | 61.85±2.98 a |
| CS+F2 | 31.59±1.02 ab | HACS+F2 | 36.51±1.70 ab | CS+F2 | 83.36±2.69 ab | HACS+F2 | 57.86±2.70 ab |
